# 4-dimensional strain imaging of the right ventricle: application in patients with severe pulmonary hypertension

**DOI:** 10.1186/1532-429X-17-S1-Q56

**Published:** 2015-02-03

**Authors:** Alessandro Satriano, Vijay Kandalam, Khalil Jivraj, Yoko Mikami, Hanna Medwid, Carmen Lydell, Naeem Merchant, Andrew G Howarth, Tracy L Elliot, James A White

**Affiliations:** 1 Stephenson Cardiac Imaging Centre, Libin Cardiovascular Institute of Alberta, University of Calgary, Calgary, AB, Canada; 2 Division of Cardiology, Department of Medicine, University of Calgary, Calgary, AB, Canada; 3 Department of Diagnostic Imaging, University of Calgary, Calgary, AB, Canada; 4 Department of Radiology, University of Calgary, Calgary, AB, Canada; 5 Queen's University, Kingston, ON, Canada

## Background

The risk stratification of patients with pulmonary hypertension (PHTN) using non-invasive techniques is challenging. 4D analysis techniques of right ventricular (RV) strain may provide unique opportunities for the identification of high-risk individuals. However, conventional strain metrics, developed for left ventricular geometry, pose limitations for RV geometry and its associated fibre orientations. In this study we examine a novel 4D strain analysis tool affording calculation of axis-independent Principal strains for the assessment of right ventricular mechanics. Patients with mild-moderate and severe PHTN were studied in comparison to a young healthy volunteer cohort.

## Methods

**Figure 1:**
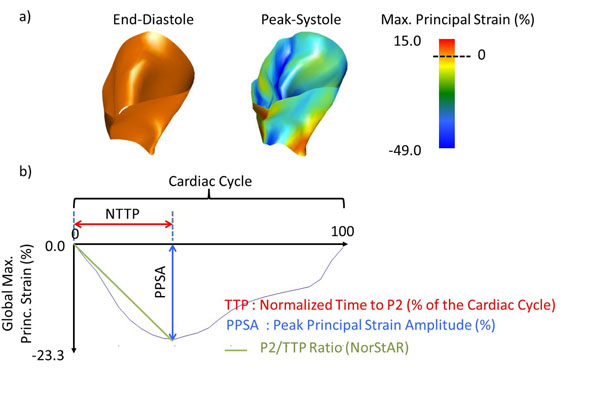
Analysis of right ventricle (RV) myocardial strain. (A) The 3D-distribution of Maximum Principal Strain (%) across the RV wall at end-diastole and end-systole. (B) Illustration of the NorStAR calculation as the ratio between the Peak Principal Strain Amplitude (PPSA) and the Normalized Time To Peak (NTTP; as a percentage of the cardiac cycle).

Fifteen patients with PHTN, defined as a mean pulmonary arterial pressure (PAP) >25mmHg at right heart catheterization, and 18 young healthy volunteers were studied. Severe PHTN was defined as a mean PAP ≥55mmHg. A standardized imaging protocol was performed using a 3T MRI scanner. Cine images were blindly analyzed for LV and RV volumes using commercial software (cvi42, Circle Cardiovascular Inc., Calgary). 4D strain analysis was performed using prototype software, generating a 3D end-diastolic mesh model of the RV and then deforming this model within a 4D displacement field, the latter contributed to by short and long-axis cine image data (Figure 1). Peak Principal Strain Amplitude (PPSA) and Normalized Time to Peak (NTTP) strain were obtained for each model node and a novel metric calculated called the "Normalized Strain Attainment Rate" (NorStAR), where NorStAR = PPSA / NNTP (Figure 1). The latter composite metric was hypothesized to represent global RV electro-mechanical recruitment.

## Results

**Figure 2:**
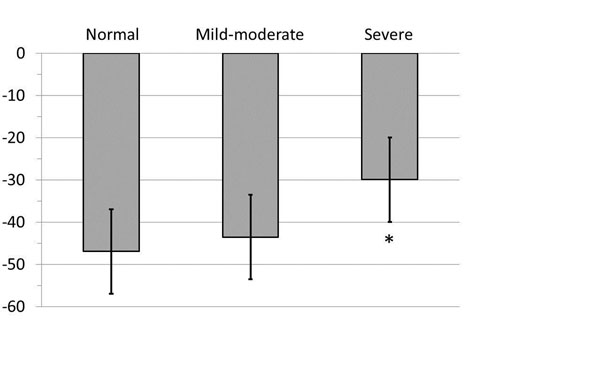
Mean values of the NorStAR strain parameter. NorStAR in healthy volunteers, mild-moderate patients, and patients with severe pulmonary hypertension, indicating a significant impairment in the severe group (* p<0.01).

The mean age of the PHTN cohort and healthy volunteers was 62.5±6.4yrs and 26.6±5.8yrs respectively. Among those with mild-moderate PHTN (n=10) the mean PAP was 39.2±9.4 (n=10) versus 62.4±9.0mmHg among those with severe PHTN (n=5). As shown in Figure 2 a significant difference in NorStAR was identified between severe PHTN and healthy controls (-30±10 vs -47±9, p=0.002), while no difference was seen for mild-moderate PHTN (-44±10 vs 47±9, P=N.S.). The capacity of NorStAR to identify patients with invasive hemodynamics of severe PHTN was incrementally assessed using ROC analysis. This achieved an AUC of 0.85 for the identification of severe PHTN with a threshold of -38 providing optimal sensitivity (80%) and specificity (60%).

## Conclusions

In this pilot study, we demonstrate the feasibility of 4D Strain analysis using Principal Strains for the characterization of RV mechanical deformation in PHTN. A novel marker, NorStAR, accurately identified patients with invasive hemodynamic evidence of severe versus non-severe PHTN and may be of value for the risk stratification of this population.

## Funding

This study was funded by the Calgary Health Trust. Dr Satriano receives support from Mitacs Canada and Medtronic of Canada, Ltd. Dr White is supported by a New Investigator Award from the Heart and Stroke Foundation of Alberta.

